# Similarities and Differences in Theory of Mind Responses of Patients With Anorexia Nervosa With and Without Autistic Features

**DOI:** 10.3389/fpsyt.2019.00318

**Published:** 2019-05-08

**Authors:** Felicity Sedgewick, Jenni Leppanen, Faith Goh, Hannah Hayward, Francesca Happé, Kate Tchanturia

**Affiliations:** ^1^Institute of Psychiatry, Psychology and Neuroscience, Psychological Medicine, King’s College London, London, United Kingdom; ^2^School of Education, University of Bristol, Bristol, United Kingdom; ^3^Department of Forensic and Neurodevelopmental Sciences, Institute of Psychiatry, Psychology and Neuroscience, King’s College London, London, United Kingdom; ^4^Medical Research Council (MRC) Social, Genetic and Developmental Psychiatry (SGDP) Centre, King’s College London, London, United Kingdom; ^5^South London and Maudsley NHS Trust EDU, London, United Kingdom; ^6^Psychology Department, Illia State University, Tbilisi, Georgia

**Keywords:** eating disorders, autism spectrum disorders, women, Theory of Mind, emotional valence

## Abstract

Theory of Mind (ToM) is the ability to understand and represent mental states of others, a skill that plays a key role in how we interact with people around us. Difficulties with ToM have been posited as an underlying mechanism for autism and implicated in difficulties faced by those with anorexia nervosa (AN). This study examined, both quantitatively and qualitatively, the responses of women between the ages of 14 and 25 years on the Frith-Happé Triangle Animations, a well-validated test of ToM. Participants were split into healthy controls (HCs), AN patients (AN), and AN patients with high levels of autistic features (AN+ASF). We found no significant quantitative differences between groups in performance on the task. Qualitatively, there were differences between groups such that AN patients, especially those in the AN+ASF group, were more focused on describing the videos than creating narratives, were more negative in their interpretations, and were much more anxious about their performance. These qualitative differences have clinical implications, including that not all AN patients with autistic features should be assumed to have difficulties with ToM.

## Introduction

Theory of Mind (ToM) is the ability to represent mental states, such as beliefs and intentions, in order to predict and explain people’s behavior (1, 2). ToM difficulties have been theorized as an explanatory mechanism for the social difficulties that are a defining diagnostic criteria for autism spectrum disorder (3). Large numbers of studies, using a range of ToM tasks, have consistently found that autistic people score lower than neurotypical counterparts in terms of their ability to extrapolate the mental states of characters [e.g., Refs. (4–6)]. A steady accumulation of evidence has shown that people with anorexia nervosa (AN) show similarities in cognitive profile to autistic individuals (7–9). Cognitive features such as poor flexibility and detail-focus (or weak “central coherence”) have been documented in both AN and autistic groups relative to healthy controls (HCs) (9, 10). People with AN and autistic people appear also to share deficits in social–emotional functioning, including ToM (11). A recent review of the literature comparing autistic people and those with AN (12) reported a number of similar difficulties, particularly in complex social situation tasks. Other meta-analyses have suggested that those with AN may have difficulties empathizing with fictional characters in ways similar to those seen in autistic people (13). Cognitive and social–emotional difficulties have been suggested to fuel the illness progression in AN by increasing isolation and making it difficult to see the big picture or change their thinking around food and exercise (14).

Although there is a solid body of research suggesting links between autism and anorexia going back to the 1980s (15, 16), most research to date on ToM in AN has not considered the potential influence of autistic features. This means that those differences and difficulties identified in previous work cannot confidently be associated with AN itself, rather than co-occurring autistic features—something that is an issue, as there is evidence that up to 23% of women with AN may also be autistic (17).

Therefore, we aimed to investigate whether AN patients who reported high levels of autistic features and those who reported low levels of autistic features differed in ToM ability, and also whether their ToM performance differed from that of HCs, either quantitatively or qualitatively. We expected that those with high levels of autistic features would perform poorly on ToM tasks, similarly to autistic participants in other studies, but that AN participants with low levels of autistic features would perform similarly to HC participants.

## Methods

### Participants

Data from 57 women between 14 and 25 years old were included in this analysis. Participants with AN and high levels of autistic features [scoring above 6 on the Autism Quotient-10 item version (AQ-10)] were first identified from a larger dataset (BEACON Study, MRC-MRF MR/R004595/1) of 171 participants, resulting in 17 participants meeting criteria. Matched samples of HCs and those with AN and low autistic traits were then identified from within the same dataset and included in this study.

Three groups [HCs, patients with AN with low levels of autistic features, and patients with anorexia and high levels of autistic features (AN+ASF)] were matched in terms of age and IQ, and each group had a similar ethnic makeup (see **Table 1**). There was a significant difference between the HC participants and all AN participants on BMI, all *p’*s < 0.001, but no difference between the AN and AN+ASF groups. Participants were recruited from a range of clinical and community sites across London under ethical approval from the London-Surrey Research Ethics Committee (17/LO/2071). Written informed consent was obtained from all participants, and written informed consent was obtained from the parents of all participants under the age of 16. All AN participants had current clinical diagnoses of AN according to the criteria of the *Diagnostic and Statistical Manual*—5th edition (3).

**Table 1:** Demographic information about participants by group.

	HC	AN	AN+ASF
n	20	20	17
Age			
Range	14.24–24.21	14.43–25.06	14.00–23.26
M (SD)	19.16 (2.73)	19.41 (3.40)	18.62 (2.51)
IQ			
Range	101.27–127.70	93.83–125.22	102.92–115.31
M (SD)	112.09 (7.64)	110.71 (7.86)	109.22 (3.57)
BMI			
Range	18.26–26.81	16.00–23.43	14.98–25.16
M (SD)	21.54 (2.67)	18.04 (1.80)	18.59 (2.83)
Ethnicity			
White n (%)	15 (75)	19 (95)	16 (94.12)
Black n (%)	2 (10)	0 (0)	0 (0)
Asian n (%)	1 (5)	1 (5)	1 (5.88)
Latinx n (%)	1 (5)	0 (0)	0 (0)

HC, healthy control; AN, anorexia nervosa patients; AN+ASF, AN patients with high levels of autistic features.

To investigate the impact of autistic features, the AN group was split into those with high-autistic (AN+ASF) and low-autistic features (AN), as measured on the AQ-10 (18). Those who scored 6 or more on the AQ-10 were categorized as AN+ASF, using the cutoff for likely autism suggested by the authors of the measure (18), and those with a score of 3 or less were categorized as AN only. We did not include participants who scored 4 or 5 in this study due to their being close to the cutoff score, as they may be among those women who are “missed” by diagnostic measures due to presenting in a non-stereotypical manner (19). HC participants were all subject to a screening call prior to taking part to ensure that they had no eating disorder past or present, and all scored less than 2 on the AQ-10 and therefore were considered a valid comparison group, without either AN or autism.

### Measures

*AQ-10*: The Autism Quotient-10 item version (18) is a 10-item questionnaire assessing autistic symptomatology. Participants respond on a four-point Likert scale, from “strongly agree” to “strongly disagree,” and items are scored either 1 or 0, depending on the direction of the endorsement. This results in a maximum score of 10 for the measure, and 6 is used as the threshold to indicate potential autism (18).

*Theory of Mind*: The Frith-Happé Triangle Animations (20, 21) are a series of 10 short silent animations (each 30–40 s long) showing two triangles moving. In two videos, the triangles move at random and do not interact. In four videos, the triangles move in a simple or goal-oriented manner, for example, pushing each other back and forth. In the other four videos, the triangles move in a way that can be interpreted as a complex interaction, such as one triangle encouraging the other to leave an enclosure. These three categories of video—Random, Goal-Oriented, and Complex/ToM—are designed to elicit different levels of ToM description from participants, who are asked to narrate the videos as they appeared on screen in line with European Autism Interventions - A Multicentre Study for Developing New Medications (EU-AIMS) methodology (22). Answers are then scored 0–2 for Accuracy and 0–2 for Mental State Terms and summed for each video type. Transcripts of participant responses allow for both quantitative and qualitative analysis. The Frith-Happé Triangle Animations have previously been used to assess ToM ability in clinical groups including autism (e.g., 23) and anorexia (24, 25). The narrative responses were recorded and transcribed for further quantitative and qualitative analysis. Transcription was conducted by one of the authors (FS) who is a native English speaker and was checked for reliability by two other authors (JL and FG). Any disagreements regarding the transcription were brought to the whole team for discussion.

*EDE-Q*: The Eating Disorder Examination Self-Report (26)-Questionnaire is a 36-item self-report questionnaire assessing eating disorder psychopathology over the past 28 days. Suggested clinical cutoff for the EDE-Q is 2.3 (27).

*HADS*: The Hospital Anxiety and Depression Scale (28) is a 14-item self-report questionnaire assessing levels of anxiety and depression over the past 2 weeks. Suggested clinical cutoff for the HADS is 8 or above on either subscale or 10 or above on the subscales combined (29).

### General Procedure

Participants were all seen at the university as part of a larger study (BEACON Study, MRC-MRF MR/R004595/1). Participants completed demographic information, the EDE-Q, and the HADS as part of online questionnaires. The larger testing session lasted approximately 3 h, including an autism assessment, a range of neurocognitive tests (including the Frith-Happé Triangle Animations), and a structural and functional MRI scan.

### Data Analysis

Quantitative data were analyzed using R (R Core Team). Group differences in clinical and demographic characteristics were assessed with ANOVA, and Hedges’ g was calculated to estimate the effect size. Group differences in ToM task accuracy and mentalizing ability were examined using Poisson regression. Finally, we also explored whether performance on the ToM task was related to eating disorder psychopathology, anxiety and depression, or body mass index (BMI) using Spearman’s correlation tests. Due to the large number of exploratory correlation analyses, the p-threshold was adjusted for multiple comparisons using the false discovery rate with q = 0.05. P-value less that 0.004 was considered significant.

Qualitative data were collected by recording the spoken responses of participants to the Frith-Happé Triangles and transcribing these verbatim. Thematic analysis of the transcripts was conducted by two authors, one acting as first coder (FS) and the second (FG) carrying out reliability coding of 20% of the transcripts. Both the first and second coder conducted the thematic analysis blind to both group and codes, so that the second individual was not aware of the themes the first had identified, and the two authors then met to discuss and agree on the results. There were no notable differences between the themes the two authors found in the participants transcripts, and the themes presented below are their consensus coding.

## Results

### Quantitative Analyses

#### Self-Report Questionnaires

There were significant differences between the groups on EDE-Q Global score, HADS Anxiety, and HADS Depression (see **Table 2** for scores). *Post hoc* t-tests revealed that both AN and AN+ASF groups scored significantly higher than HC participants on the EDE-Q Global score, HADS Anxiety, and HADS Depression. There was no significant difference between the AN and AN+ASF groups on the EDE-Q, *t*(35) = −1.85, *p* = 0.07. There were significant differences between the AN and AN+ASF groups on HADS Anxiety, *t*(35) = −4.21, *p* < 0.001, and HADS Depression, *t*(35) = −3.81, *p* = 0.001, such that AN+ASF participants were more anxious and more depressed than AN participants. There were no correlations between AQ-10 and BMI in the HC group (ρ = 0.13, *p* = 0.581), AN group (ρ = 0.00, *p* = 1.00), or the AN+ASF group (ρ = −0.19, *p* = 0.475). There were no correlations between AQ-10 score and EDE-Q score in the HC group (ρ = −0.02, *p* = 0.949), AN group (ρ = 0.17, *p* = 0.485), or AN+ASF group (ρ = −0.39, *p* = 0.117). There was also no significant correlation between AQ-10 scores and HADS scores within the HC group (㌸ρ = −0.04, *p* = 0.862), the AN group (㌸ρ = −0.022, *p* = 0.355), or the AN+ASF group (㌸ρ = 0.11, *p* = 0.675).

**Table 2:** Scores on mental health measures by group.

	HC	AN	AN+ASF	F-statistic *p-value*	Hedges’ g ES [95% CI]
AQ-10					HC vs. AN: −0.76, [−1.42, −0.09] HC vs. AN+ASF: −5.58 [−7.06, −4.10] AN vs. AN+ASF: −5.48 [−6.93, −4.02]
Range	0–2	1–2	6–10	*F*(2) = 268.03,	
M (SD)	1.00 (1.00)	2.00 (1.00)	7.00 (1.00)	*p* < 0.001*	
EDE-Q Global					HC vs. AN: −2.17, [−2.98, −1.36] HC vs. AN+ASF: −3.03, [−4.01, −2.05] AN vs. AN+ASF: −0.60, [−1.28, 0.09]
Range	0–0.73	0.28–5.12	0.35–5.12	*F*(2) = 38.37,	
M (SD)	0.23 (0.20)	2.63 (1.52)	3.54 (1.44)	*p* = < 0.001*	
HADS Total					HC vs. AN: −1.11, [−180, −0.43] HC vs. AN+ASF: 2.48, [−3.37, −1.59] AN vs. AN+ASF: −1.48, [−2.23, −0.72]
Range	1–21	3–27	14–42	*F*(2) = 32.29,	
M (SD)	8.15 (5.76)	14.65 (5.67)	24.12 (6.74)	*p* = < 0.001*	

AQ-10, Autism Quotient-10 item version; EDE-Q, Eating Disorder Examination Self-Report Questionnaire; HADS, Hospital Anxiety and Depression Scale.

*Denotes a significant result.

#### Theory of Mind: Accuracy

On Accuracy, there was no significant difference between the three groups on either the Random, the Goal-Oriented, or the ToM animations (see **Table 3** for scores).

**Table 3:** Accuracy and Mental State Language scores by group and video type.

	HC	AN	AN+ASF	*X^2^*-statistic *p-value*	Cramer’s *V*
Accuracy					
Random Range	2–4	0–4	0–4	*X* ^2^(2) = 0.65,	0.08
Median (IQR)	3.0 (2.0)	3.0 (2.0)	4.0 (1.5)	*p* = *0.721*	
Accuracy					
Goal-oriented Range	1–9	2–7	3–7	*X* ^2^(2) = 0.45,	0.06
Median (IQR)	5.0 (2.0)	6.0 (1.0)	5.0 (1.5)	*p* = *0.799*	
Accuracy					
Complex Range	2–7	1–6	0–7	*X* ^2^(2) = 1.03,	0.10
Median (IQR)	4.0 (0.5)	4.0 (2.0)	3.0 (2.0)	*p* = *0.599*	
Mental State					
Random Range	0–1	0–2	0–1	*X* ^2^(2) = 2.30,	0.14
Median (IQR)	0.0 (0.0)	0.0 (1.0)	0.0 (0.0)	*p* = *0.317*	
Mental State					
Goal-oriented Range	0–2	0–4	0–3	*X* ^2^(2) = 1.12,	0.10
Median (IQR)	1.0 (2.0)	0.0 (2.0)	0.0 (1.0)	*p* = *0.572*	
Mental State					
Complex Range	0–7	0–7	0–6	*X* ^2^(2) = 5.89,	0.28
Median (IQR)	3.0 (1.5)	3.5 (2.0)	2.0 (3.0)	*p* = *0.053*	

Italicisation is a convention of the field.

#### Theory of Mind: Mental State

On Mental State, there were no significant differences between the three groups in any of the video conditions (Random, Goal-Oriented, Complex, Total; see **Table 3** for scores).

#### Clinical Measures and Theory of Mind

We explored whether levels of eating disorder behaviors, anxiety and depression, autistic features, and BMI had an impact on either accuracy or on number of mental state terms, negative, and positive terms participants used in their narrations. Due to there being no significant group differences between the HC, AN, and AN+ASF, all participants were included in the exploratory correlation analyses. There were no significant correlations between Accuracy or Mental State Language and any clinical measures across all participants (see **Table 4**).

**Table 4:** Correlations between self-reported mental health and Theory of Mind.

	Accuracy	Mental State Language	Negative terms	Positive terms
EDE-Q Total	ρ = −0.20, *p* = 0.135	ρ = −0.19, *p *= 0.155	ρ = −0.01, *p* = 0.932	ρ = 0.11, *p* = 0.409
HADS Total	ρ = −0.18, *p *= 0.190	ρ = −0.17, *p* = 0.195	ρ = −0.01, *p* = 0.949	ρ = 0.03, *p* = 0.813
AQ-10 Total	ρ = −0.09, *p* = 0.518	ρ = −0.13, *p* = 0.320	ρ = −0.12, *p* = 0.354	ρ = −0.06, *p* = 0.614
BMI	ρ = 0.23, *p *= 0.091	ρ = 0.29, *p* = 0.030	ρ = 0.22, *p* = 0.107	ρ = 0.21, *p* = 0.125

#### Qualitative Analysis

**Figure 1:**
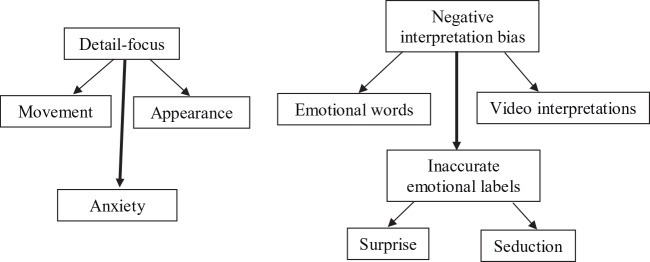
Qualitative map of themes arising from narrations of the Frith-Happé Triangle Animations.

Qualitatively, there were notable differences between the three groups. These differences can be characterized as coming under the themes of *detail focus*, *negative interpretation bias, inaccurate emotional labels*, and *anxiety* and are visualized in **Figure 1**.

*Detail focus*. The most obvious difference between the patients and the HC participants was their different level of focus on the detailed geometry and *movement* in the videos. AN patients, particularly those with high AQ scores, gave greater focus to precisely where items were on the screen, for example, saying “the smaller box with the open side is now in the bottom right hand corner” (AN+ASF; Chasing) or “the little triangle is moving in a clockwise direction and the bigger triangle in an anti-clockwise direction” (AN; Bouncing). While some HC participants also described where the triangles were throughout the videos, these descriptions tended to relate their positioning to each other—“the little one is above the big one” (HC; Floating). In contrast, AN participants often described the triangles independently of each other, even in interaction videos—“the little one is in the box and the big one is going in and out of the box” (AN+ASF: Coaxing).

AN participants also placed more emphasis on describing the *appearance* of the triangles, rather than creating a narrative around their actions. For example, the patient group said things like “the lines keep getting smoother” (AN+ASF; Bouncing), “they are drawn in graphite” (AN; Surprise), and “they’ve squashed so they aren’t isosceles triangles anymore, they’re more like obtuse triangles” (AN+ASF; Fighting). While some HC participants did describe the layout of the screen—“there’s a smaller box with an open side” (HC; Coaxing)—this was far less common and was usually instrumental to describing the action, rather than being a standalone comment. In the above example, the sentence was finished by adding “and the triangles are inside it” (HC; Coaxing), whereas AN participants tended to focus on describing the physical appearance of the objects on the screen rather than providing a narrative.

*Negative interpretation bias*. Another key qualitative difference between the groups was the prevalence of *negative emotional words*. Both AN and AN+ASF participants used far more negative terms and gave more negative interpretations of the triangles’ movements than HC participants, describing them as “crying,” “fighting,” and “angry” more often than HC participants. They gave more involved descriptions of these negative emotions and cognitions than for positive ones—“he’s hiding in the corner shaking ‘cos he’s scared and trapped” (AN; Seduction) compared to “they’re happy they’ve found each other” (HC; Surprise). AN+ASF participants were most likely to give these negative interpretations, with AN participants representing a “mid-point” between the AN+ASF and HC groups in terms of the negative emotions they assigned to the triangles. Rather than the negative emotions common among AN+ASF participants, AN and HC participants mostly used emotionally neutral terms—“wants to get out,” “is looking for the other one,” or “tries to get in the box.” These terms all imply a mental state or goal of the character, without giving much emotional weight to those mental states and desires.

Beyond the individual negative terms used by the patient group, the AN+ASF group gave much more *negative interpretations of the videos* overall. For example, one participant described the Coaxing scenario (where the big triangle pulls the little one out of the “house” to spin and play in the “garden”) as the two triangles fighting and the “small one crying in the corner because it was scared,” then spinning because it was “scared and confused.” This is both inaccurate and a negative way of seeing the situation, which is supposed to show two characters having fun. More AN+ASF participants gave these unhappy interpretations of either neutral or positive scenarios compared to AN and HC participants who were more positive.

As a specific example of this negative interpretation, the “Surprise” video is supposed to represent a game of “knock and run”—the larger triangle is inside the “house” by itself, and then the smaller one enters and bumps several times on the “door” (“knocking”). The larger triangle opens the door (side of the box), comes part way out, and bends from side to side, “looking” for the one who knocked, while the small one “hides” behind the door. The large triangle goes back inside, and the sequence is repeated. On the third time of “knocking,” the small triangle “surprises” the large one by coming out from behind the door, and then the two triangles enter the box together and spin (intended to represent hugging or being pleased to see each other).

The authors of the animations expected participants to see this as a game of knock and run between a grandmother and grandson (or similar characters), and to give answers relating to the game and the small triangle surprising or tricking the large triangle. Instead, AN participants frequently interpreted the scenario negatively. Common narratives were that the large triangle was “trapped” in the box and “can’t get out,” or that the small triangle was “locking it in,” “turning a switch to keep it in,” or that the large triangle “is locking [the small one] out when it wants to get in.” When they used ToM terms to describe how the triangles felt about the scenario, they used terms associated with negative emotions such as “sad,” “worried,” and “feels unwanted,” rather than the positive emotions of surprise and joy that the authors intended to be seen in the video. This was true for all AN participants, rather than being affected by ASF status.

*Inaccurate emotional labels.* Linked to this negative interpretation bias, participants often misinterpreted the emotional valence of videos, seeing them as negative when they were neutral or positive in nature. Participants generally used more negative than positive emotional labels when narrating the videos, and the negative words they used were more intense than the positive terms, i.e., “[the triangle is] shaking and scared” or “[it looks like it’s been] murdered” compared to “[the big one is] pleased” and “[they’re having] fun.”

Interestingly, the Seduction scenario was also interpreted very differently by participants than intended according to the scoring manual. The interpretation given in the scoring manual is that of a princess tricking a guard into letting her escape—a situation that could be interpreted negatively, although there is a happy ending. This video, which would seem to lend itself to the negative interpretations shown by AN+ASF participants elsewhere, did not elicit the same kind of strong negative responses. Instead, AN+ASF participants tended to simply describe the geometry of this video, as discussed above. In contrast, AN and HC participants tended to describe the small triangle as “escaping” or “running away,” and the large triangle as “confused”—terms that imply mental states being attributed to the characters, even if they were presented in terms with neutral emotional valence.

*Anxiety.* Another notable pattern, possibly linked to the detail-focus discussed above, was that the patient group was much more likely than HC participants to ask questions of the examiner during the administration of the task, focused on making sure that they were getting things “right” or performing as expected. Several AN and AN+ASF participants asked questions such as “Is that what you wanted?,” “Is that right?,” or “Have I got it all?” Participants in both the AN+ASF and AN groups were notably more anxious about saying the right thing, giving the right interpretation, and simply whether they were completing the task correctly than HC participants were. This desire to give “perfect” responses, and the accompanying anxiety about not doing so, was not present in HC participants.

## Discussion

The findings of this study suggest that while there were no statistically significant quantitative differences between HC participants, AN participants, and AN+ASF participants on the Frith-Happé Triangle Animations, there are some interesting qualitative differences. This suggests that people with AN may not have significant difficulties in ToM, regardless of their level of self-reported autistic features. Although several studies have reported significantly reduced ToM in people with AN using a variety of tasks (12), more recently a few larger studies using a variety of different measures have found no group differences (24). Together, these findings suggest that women with AN may have specific social–emotional difficulties that may not extend to explicit labeling and recognition of emotions and mental states, but may rather be reflective of more subtle qualitative difficulties, such as interpretation biases.

Contrary to our hypothesis, there were no significant differences between the high-AQ and low-AQ groups in terms of Accuracy or Mental State Language, regardless of AN status. It should be pointed out, however, that scoring above cutoff on the AQ-10 is distinctly different to receiving a clinical diagnosis of being autistic, meaning that these individuals may not be as similar in performance on the task to autistic people as might be expected. Further to this, the AQ-10 is a basic screening tool and, while widely used, has been shown to be less reliable than the longer versions of the AQ, such as the AQ-50 or AQ-28 (30, 31), or other self-report questionnaires such as the Social Responsiveness Scale—2^nd^ Edition (32). However, we can be confident in this sample that the AQ-10 was reflective of autistic features rather than mental health issues, as there was no correlation between AQ-10 score and HADS score in any group.

It is also worth noting that previous research that has supported the effectiveness of the Frith-Happé Triangle Animations as a measure of ToM ability, and as revealing differences between autistic and neurotypical groups, has had mainly male participants. The non-significant quantitative findings in this study may therefore actually represent a gender difference according to the scoring method that focuses on accuracy in explicit labeling and recognition. What little evidence there is as to gender differences in ToM ability suggests that girls score more highly than boys (20, 21, 33). Among neurotypical children, one study found that 3- to 5-year-old non-autistic girls scored better on ToM tasks than non-autistic boys (34) on the Sally-Anne False Belief task, and this gender difference is also seen in late childhood (35). Most work that finds autistic people struggle with ToM has used majority-male participant samples, as is the case for much autism research (36). This means that we know very little about ToM ability in autistic girls and women, or in girls and women with high levels of autistic features. There is, to date, little research on the ToM skills of older girls and women with eating disorders, regardless of their autism status, a lack that this paper seeks to somewhat redress. Future work should seek matched groups of males with AN and autistic males and females without AN in order to establish the true nature of the similarities and differences in ToM in AN and any potential relationship to autistic features. It is also worth noting that all participants in this sample had relatively high IQ scores, something that may ameliorate difficulties with ToM traditionally seen among autistic individuals.

The findings of this research suggest that the Frith-Happé Triangle Animations (20) may not be the most sensitive measure of ToM to use in a female neurotypical population, similar to other research findings (37). All women in this study scored similarly on both Accuracy and Mental State, regardless of levels of self-reported autistic features. Also, most participants did not create story-like narratives for the videos, instead describing the screen and the movements of the triangles, something that may have been induced in part by the direction to describe the video as it was happening. This suggests that although the task seeks to examine ToM skill—looking at the intentions and motives assigned to the two triangles—the administration instructions do not explicitly ask for this, and therefore, some participants may not show their actual level of ToM skill. While some participants did create stories and use a range of ToM terms, most did not create complex narratives, instead giving individual mentalizing terms or inconsistently using designating the triangles as characters.

The qualitative findings of this study echo those of other work that has suggested similarities between the social and cognitive experiences of autistic people and patients with AN. The differences in the negative interpretations of the scenarios between HC/AN participants and AN+ASF participants were clear. That AN+ASF participants generally use more negative terms and give more negative interpretations to the Frith-Happé Animations aligns with previous findings of negative interpretation bias in AN (38–40). This is further evidence that AN patients with co-occurring autistic traits may need extra or individually tailored support in their treatment programs, as social support can be crucial to recovery (41–43), but if they are consistently interpreting their social experiences negatively, they may be struggling to access that support. Difficulties with negative interpretation are potentially further exacerbated by the higher levels of depression and anxiety in the AN group, as individuals with depression (44, 45) and anxiety (46, 47) have been shown to interpret situations negatively. Therefore, patients who have co-occurring AN, depression, and autism may be particularly negative in their views of their social experiences, which creates a self-fulfilling cycle where they interpret a situation negatively and withdraw or react inappropriately; therefore, those around them are less supportive, reinforcing the idea that they do not have social support, and giving the impression that their initial negative interpretation was correct. This interpretation is supported by work showing that those who are “affective deviants,” i.e., who react in non-normative ways to social situations, are judged more negatively by those they interact with and are more likely to be avoided by others (48).

Other qualitative work examining emotions in anorexia found that patients had difficulties with emotional expression and negative emotions (49), all of which are also seen among autistic people (50–52). Similarly, patients with AN have been shown to have difficulties with their friendships and social relationships, which predate the onset of their illness (53, 54), and challenges with social relationships and imagination are a key diagnostic feature of autism (3). Importantly, recent research has shown that these difficulties are present for autistic women and girls (55–57), meaning that these experiences are directly comparable to those of female AN patients.

The focus of AN participants, particularly AN+ASF participants, on describing the layout of the screen and the movements of the triangles provides qualitative evidence of the detail-oriented processing and weak central coherence that has been previously seen in both those with AN (10, 58, 59) and those on the autism spectrum (60–62). It may be that individuals at the intersection of the two conditions, potentially represented by the AN+ASF group, would have that much more of a focus on details rather than the overall narrative. Knowing that this is a potential cognitive profile, as with the tendency to negative interpretation bias, has clinical implications. Detail-oriented processing over global processing is a known factor in many treatment programs designed to support AN recovery, but if a patient also has high levels of autistic features, they may find it particularly difficult to move on from this thinking style, as it may be linked not only to their illness but also to their underlying neurotype. This means that clinical teams would need to adapt treatment approaches to their particular needs and may need to reframe the ways in which these approaches are presented (63).

The fact that AN patients asked far more questions than the HC participants points to the much higher anxiety levels of the patient group. These higher anxiety levels are borne out in the quantitative as well as qualitative data, with AN and AN+ASF participants being more anxious than HC participants. There is a wealth of research evidencing higher anxiety levels in AN patients (64, 65) and autistic people (66–68). The emphasis in these questions on whether participants were “doing the right thing” or “giving the right answer” suggests that AN and AN+ASF participants were especially anxious about their performance on the task rather than how to complete it, an attitude that may be linked to the high levels of perfectionism seen in those with AN (69–71).

## Limitations

While there were limitations to this study, such as the small sample size, the number of participants is sufficient for the analyses conducted, as shown by the range and consistency of the group differences identified. In the quantitative analysis, there is, however, the possibility of type II errors when working with a small sample size, as there may be insufficient power to detect a true effect leading to a negative finding. Therefore, larger studies are needed before firm conclusions regarding ToM difficulties in people with AN and ASF can be drawn. Future quantitative studies may also benefit from including a larger sample to assess the impact of eating disorders psychopathology, illness stage, and sub-type along with other mental health measures on ToM scores to gain a more holistic picture of social–emotional difficulties in AN.

Another issue is the potential lack of sensitivity of the AQ-10, but it is widely used as a screening measure both clinically (17, 72) and in research (18, 73, 74). The fact that the use of the AQ-10 cutoff did not make a statistically meaningful difference to the outcome measures also suggests that the AQ-10, while quick to administer, may not be the most effective screening tool in a clinical setting, and therefore it may be worth clinical teams taking more time to use more thorough measures. In future work, it will be crucial to have a comparison group of people with AN who also have clinician-verified autism diagnoses, and a comparison group of autistic people without AN, to more fully examine the impact of autistic features on social–emotional difficulties in AN and place these in the context of autism itself. The all-female nature of this sample is also a limitation of the study. While most ToM work has had majority-male samples, as it comes from the autism field, most AN work has majority-female samples, as these are the people who are most often diagnosed with eating disorders. Conducting research with gender-balanced samples will be important in the future to redress the existing gender imbalance in both autism and eating disorder research and will allow us to more accurately evaluate and describe group- and gender-based differences in these skills.

## Conclusion

Overall, the findings of this study suggest that while there may not be quantitative differences in task performance between HCs, AN patients with low-AQ scores and patients with high-AQ scores on the Frith-Happé Triangle Animations test of ToM, the qualitative differences between the groups may have clinical implications. The findings also bring into question the assumption that everyone with high levels of autistic features will have difficulties with ToM tasks, highlighting that this may instead be a feature of autism in males rather than females.

## Data Availability Statement

The datasets generated for this study are available on request to the corresponding author.

## Author Contributions

FS and JL conducted data collection, analysis, and primary write-up of the manuscript. HH and FH contributed to initial research design and editing of the manuscript. FG contributed to data transcription and analysis, and to edits of the manuscript. KT was PI for the project, was awarded the funding which made it possible, was responsible for initial study design and contributed to recruitment and edits of the manuscript.

## Funding

All authors would like to acknowledge the MRC-MRF Fund (MR/R004595/1) for making this research possible. KT would also like to acknowledge funding from The Health Foundation (AIMS ID: 1115447).

## Conflict of Interest Statement

The authors declare that the research was conducted in the absence of any commercial or financial relationships that could be construed as a potential conflict of interest.
